# Tumor-associated epilepsy and high expression of xCT shape the proteome of IDH-wildtype glioblastoma

**DOI:** 10.1038/s41420-026-03029-7

**Published:** 2026-03-25

**Authors:** Iris Divé, Jasmin A. Schäfer, Katharina J. Weber, Ali Yavuz Çakır, Nikita A. Verheyden, Nadja I. Lorenz, Seon-Ah Chong, Pia S. Zeiner, Carmen Franiczek, Benedikt Sauer, Martin Adrian-Allgood, Hans Urban, Anna-Luisa Luger, Katharina J. Wenger, Adam Strzelczyk, Felix Rosenow, Johannes Streffer, Vincent Prinz, Daniel P. Brucker, Stefanie Dedeurwaerdere, Véronique M. André, Joachim P. Steinbach, Florian Buettner, Christian Münch, Michael W. Ronellenfitsch

**Affiliations:** 1 Dr. Senckenberg Institute of Neurooncology, University Hospital Frankfurt, Goethe University, Frankfurt am Main, Germany; 2 University Cancer Center Frankfurt (UCT), Frankfurt am Main, Germany; 3 German Cancer Consortium (DKTK), Partner Site Frankfurt/Mainz, a partnership between DKFZ and UCT Frankfurt-Marburg, Frankfurt am Main, Germany; 4 Frankfurt Cancer Institute (FCI), University Hospital Frankfurt, Goethe University, Frankfurt am Main, Germany; 5 Institute of Molecular Systems Medicine, Goethe University, Frankfurt am Main, Germany; 6 Institute of Neurology (Edinger-Institute), Goethe University, Frankfurt am Main, Germany; 7 German Cancer Research Center (DKFZ), Heidelberg, Germany; 8 Goethe University Frankfurt, Department of Medicine, Frankfurt am Main, Germany; 9 UCB Pharma, Chemin du Foriest 1, 1420 Braine-l’Alleud, Belgium; 10 Institute of Neuroradiology, Goethe University, Frankfurt am Main, Germany; 11 Epilepsy Center Frankfurt Rhine-Main, Department of Neurology, Goethe University, Frankfurt am Main, Germany; 12 Reference Center for Biological Markers of Dementia (BIODEM), Institute Born-Bunge, University of Antwerp, Antwerp, Belgium; 13 Goethe University Frankfurt, Department of Neurosurgery, University Hospital Frankfurt, Frankfurt am Main, Germany; 14 UCT Biobank, University Hospital Frankfurt, Goethe University, Frankfurt am Main, Germany

**Keywords:** CNS cancer, Epilepsy

## Abstract

Given the role of glutamate signaling in glioma-associated epilepsy (GAE) and glioma cell growth, amino acid transporters have gained attention as therapeutic targets. Here, we conducted a comparative analysis of four key transporters—xCT, CD98, EAAT2, and ASCT1—with particular emphasis on xCT, due to the availability of clinically established inhibitors. Protein expression was quantified by immunoblot in snap-frozen tissue of tumor treatment-naïve IDH-mutant and IDH-wildtype gliomas (*n* = 87) with and without GAE. Quantitative whole-cell proteomics was performed on glioblastoma (GB) from 16 patients stratified for GAE and xCT expression levels. Gliomas from patients with GAE showed significantly higher EAAT2 and ASCT1 levels. In IDH-mutant versus IDH-wildtype glioma, xCT, EAAT2 and ASCT1 were significantly upregulated. Quantitative proteomics revealed 214 significantly regulated proteins in GB with GAE. Upregulated proteins showed enrichment for Gene Ontology (GO) terms involving neurotransmitter and amino acid turnover as well as lipid metabolism. Within the epilepsy group, xCT high-expressing tumors had distinct enrichment patterns. The 231 upregulated proteins partially overlapped with proteins upregulated in the epilepsy cohort, but additionally showed enrichment in pathways related to myelination and synaptic plasticity. In the survival analysis (*n* = 87), xCT expression and epilepsy did not affect patient survival in either IDH-mutant or IDH-wildtype tumors. Our findings highlight the role of amino acid transporters in GAE. The proteome analysis reveals distinct patterns in GB with epilepsy and also underscores the influence of xCT expression on the tumor proteome, which could inform the development of targeted anti-seizure medication.

## Introduction

Epilepsy is a frequent co-morbidity in glioma patients affecting up to 80% of patients during the course of their disease [1]. As seizures, and status epilepticus in particular, can lead to clinical worsening or tumor treatment delay [2, 3], effective anti-seizure medication (ASM) is a crucial element of the clinical management of brain tumor patients.

**Fig. 1:**
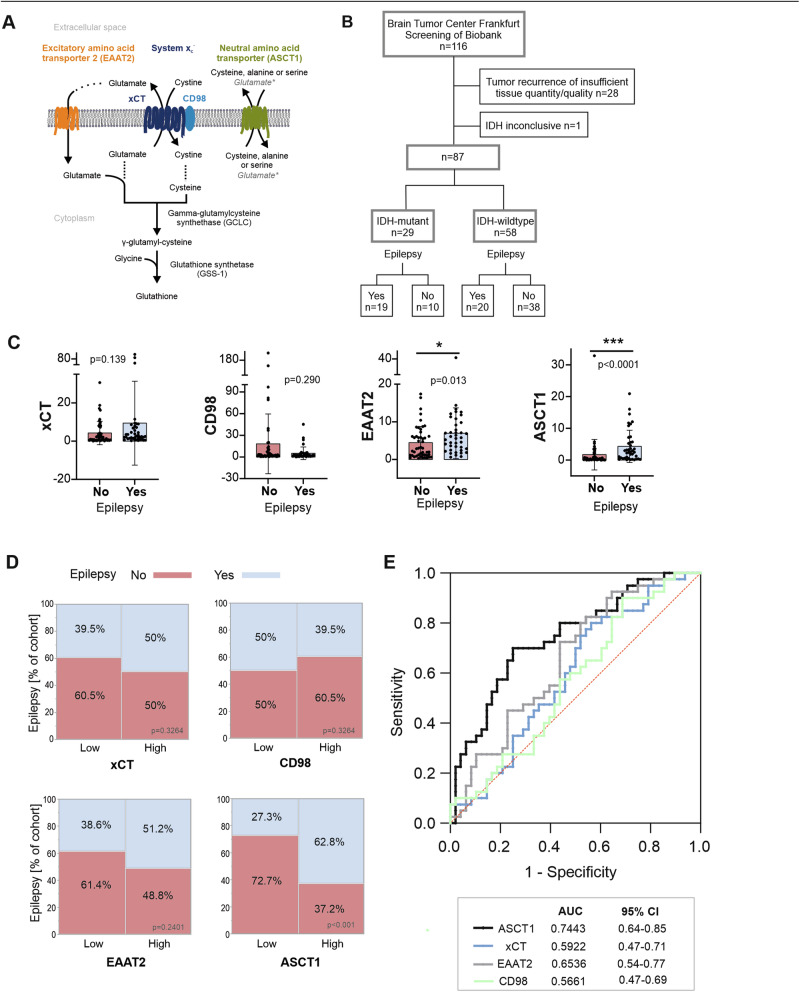
Protein expression of xCT, CD98, EAAT2 and ASCT1 in glioma with vs. without epilepsy. **A** Schematic overview of the membrane glutamate transport and glutathione synthesis. Glutamate release is primarily facilitated through system x_c_^−^ which consists of a catalytic subunit, xCT and a regulatory subunit, CD98. Uptake of glutamate is mediated by the excitatory amino acid transporter 2 (EAAT2; synonym solute carrier family 1 member 2 (SLC1A2)) and the alanine/serine/cysteine/threonine transporter 1 (ASCT1), also designated neutral amino acid transporter A. *ASCT1 displays pH-dependent substrate specificity and transports glutamate at low pH levels. **B** Consort diagram of the study. **C** Protein expression levels of xCT, CD98, EAAT2, and ASCT1 as assessed by immunoblot in relation to epilepsy at first diagnosis. Values represent protein expression normalized to actin (*n* = 87, bars = mean, error bars = SD; unpaired, two-tailed *t*-test). For uncropped membranes of immunoblots, please refer to Supplementary Fig. 3. **D** Distribution of epilepsy in the low vs. high-expressing cohort of the indicated protein; classification of either low- or high-expressing was based on the median of the entire cohort. Values are given as a percentage of the respective subcohort. P-values correspond to Pearson’s chi-squared test. **E** Receiver operating characteristics (ROC) curve for xCT, CD98, EAAT2 and ASCT1.

In glioma, the occurrence of seizures has been linked to increased levels of extracellular glutamate in the tumor and peritumoral tissue. High glutamate levels enhance signaling through NMDA receptors, which, in turn, leads to increased calcium influx on the one hand, and degradation of an inhibitory GABA_B_ receptor subunit on the other hand [4–6]. Moreover, recent findings show that glutamate is a key mediator in neuron-to-glioma synapses that drive both neuronal hyperexcitability and glioma progression [7]. As a result, amino acid transporters have attracted interest as therapeutic targets with the ability to control both seizure activity and glioma progression [8]. Among these, the glutamate transporter xCT (Fig. 1A) has received particular attention, in part because it can be inhibited by sulfasalazine, a pharmacological agent already approved for clinical use [8, 9]. In animal models, high xCT expression correlated with seizures that were suppressed by sulfasalazine [10]. In line with this, MR spectroscopy studies of glioma patients positively correlated xCT expression with glutamate release, which was counteracted by oral sulfasalazine [11]. Prognostically, high xCT expression has been linked to shorter overall survival both in vivo and in a retrospective study of glioma patients [11]. While xCT and its regulatory subunit, CD98 (also known as 4F2hc), regulate glutamate release, glutamate uptake is facilitated mainly through the excitatory amino acid transporter 2 (EAAT2; synonym solute carrier family 1 member 2 (SLC1A2) [11]. Pharmacologically, it can be targeted by PPARγ agonizts like pioglitazone and glutamate modulators such as riluzole or its prodrug, troriluzole [12]. In vitro, EAAT2 inhibition with pioglitazone decreased both glutamate levels and glioma viability [13], while treatment with riluzole enhanced the effects of temozolomide both in vitro and in vivo [14] and potentiated radiation-induced cytotoxicity [15]. At low pH levels, when the function of EAAT transporters is impaired, glutamate is transported by the alanine/serine/cysteine/threonine transporter 1 (ASCT1) [16, 17]. Recent evidence from RNA sequencing data suggests that the expression of ASCT1 may serve as a predictor of postoperative seizure occurrence in glioma patients [18].

Previous studies have largely examined individual amino acid transporters, rather than their collective function within the context of GAE. Moreover, some of the (retrospective) clinical studies originate from the period preceding the current WHO classification of CNS tumors, limiting their interpretability. To address these gaps, the present study characterizes dysregulation of the glutamate transport network through a comparative analysis of xCT, CD98, ASCT1 and EAAT2 in a cohort of molecularly classified gliomas. Given the strong evidence base for xCT and the availability of a clinically approved inhibitor, we place particular emphasis on this transporter and use whole-cell proteomics to identify protein clusters linked to both GAE and xCT expression, thereby providing a comprehensive framework for biomarker discovery.

## Results

### Expression of amino acid transporters in relation to glioma-associated epilepsy and IDH status

We identified 87 glioma cases, of which sufficient quality snap-frozen tissue from tumor resection was available (Fig. 1B). IDH-mutant tumors comprised IDH-mutant astrocytoma WHO grade 3 (*n* = 18) or grade 4 (*n* = 6) and oligodendrogliomas WHO grade 3 (*n* = 5). We assessed protein expression levels of xCT, EAAT2, CD98, and ASCT1 by immunoblot (Fig. 1C), as no reliable antibody for xCT immunohistochemistry analysis was available. Notably, we also evaluated the current lot of an already published xCT antibody in brain sections from xCT KO mice, demonstrating that the antibody is not specific to xCT (supplementary Fig. 1). In the total glioma cohort, expression of EAAT2 and ASCT1 was significantly higher in patients with epilepsy. The epilepsy cohort exhibited a trend toward increased protein expression of xCT; however, these values did not achieve statistical significance (*p* = 0.139). CD98 was expressed inversely, showing lower protein levels in tissue obtained from patients with epilepsy (*p* = 0.290). IDH-mutant gliomas had significantly higher levels of xCT, EAAT2, and ASCT1, and lower expression levels of CD98, compared to IDH-wildtype GB (supplementary Fig. 2A). These patients showed a higher incidence of seizures at the time of diagnosis (Supplementary Fig. 2B).

**Fig. 2:**
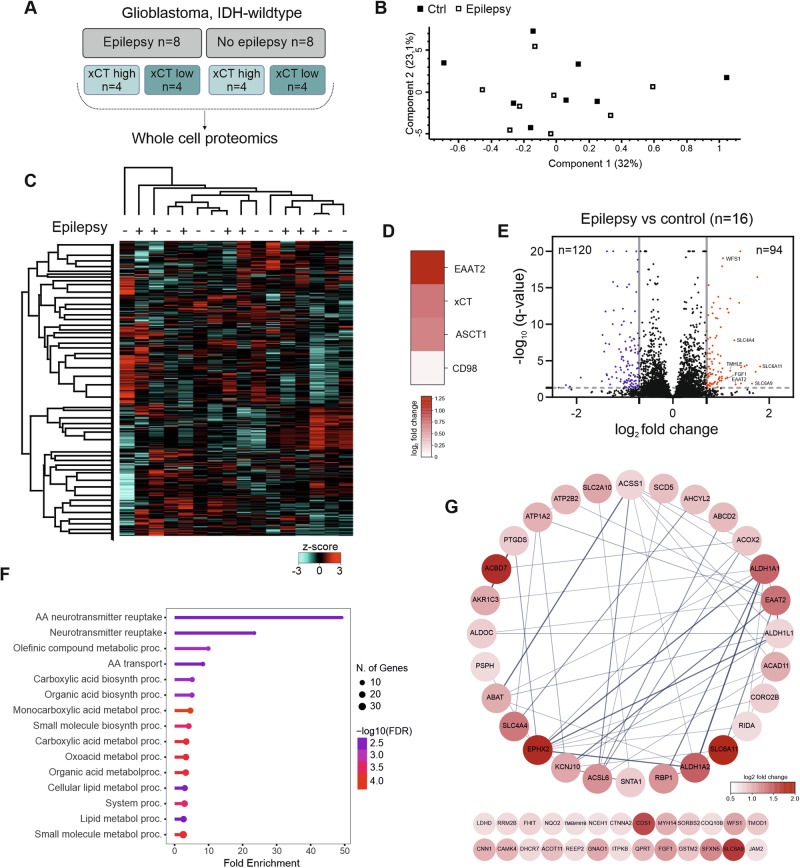
Whole-cell proteomics of glioblastoma samples with and without epilepsy. **A** Overview of sample selection. **B** Principal component analysis of all 16 samples. **C** Heatmap and unsupervised hierarchical clustering; depicted are z-score values. **D** Heatmap displaying the *q*-value-independent fold change of the indicated proteins in the epilepsy vs. non-epilepsy group. **E** Volcano plot showing fold change versus *q*-value for the epilepsy vs. the non-epilepsy group. Numbers indicate the total amount of significantly regulated proteins (*q*-value < 0.05, fold change ≤−0.07 or ≥0.07). Annotated proteins are encoded by genes in which mutations cause epilepsy. *Q*-values > 20 were set to *q* = 20 for plotting; for original *q*-values, please refer to Supplementary Table 1. **F** Protein enrichment analysis of significantly upregulated proteins (*q*-value < 0.05, fold change ≥ 0.07); displayed are GO-BP terms (*q*-value < 0.05), pathways are sorted by fold enrichment. **G** STRING analysis of the 54 proteins represented in GO-BP terms in (**F**).

Based on the median expression level of the entire cohort, we classified samples as low- or high-expressing for each of the four proteins (Fig. 1D). In the contingency analysis, high ASCT1 expression was significantly associated with seizures (Pearson’s chi-squared test *p* < 0.001). It should be emphasized that high ASCT1 expression was mainly observed in IDH-mutant tumors (Supplementary Fig. 2C). For EAAT2 and xCT, we observed similar trends, although values did not reach statistical significance. Again, CD98 stood out in that the lower-expressing cohort had a higher percentage of epilepsy. In the receiver operating characteristic (ROC) curve analysis (Fig. 2E), ASCT1 demonstrated the strongest diagnostic performance (area under the curve (AUC) 0.7422). EAAT2 showed a moderate discriminatory ability (AUC 0.6536) while xCT exhibited a poor predictive performance (AUC 0.5922).

To corroborate the immunoblot results, we examined publicly available independent mRNA expression datasets from IDH mutant (supplementary Fig. 2D) and IDH wildtype gliomas (supplementary Fig. 2E). In the IDH mutant cohort, we found a statistically significant upregulation of *SLC1A4*, which encodes ASCT1, and *SLC1A2*, which encodes EAAT2. For *SLC7A11* (encoding xCT), no clear regulation was detected. In line with our immunoblot results, *SLC3A2* (encoding CD98) showed a non-significant downregulation. The analysis of IDH wildtype gliomas differed in that it showed a distinct and statistically significant upregulation of *SLC7A11* in the seizure group, whereas the upregulation of *SLC1A2* and *SLC1A4* was present, but not significant. Again, *SLC3A2* showed a non-significant downregulation in the seizure group.

### Proteomic characteristics of IDH-wildtype glioblastoma with glioma-associated epilepsy

By employing quantitative whole cell proteomics, we aimed to identify protein networks linked to the occurrence of epilepsy in GB on the one hand, and defined xCT expression levels on the other. We selected samples based on epilepsy status and either high or low xCT expression level. To ensure clear group separation, we selected only samples ranked within the top 10% of either high or low xCT expression, as previously assessed by immunoblot (Fig. 2A).

The tandem mass tag (TMT) multiplexed-labeling approach provided 7903 proteins expressed across all samples for comparative analyses. Global approaches to assess differential protein expression, including principal component analyses and unsupervised clustering, revealed no apparent differences in the proteome of GB with epilepsy compared to the non-epilepsy cases (Fig. 2B, C). The unfiltered analysis of the data corroborated an over-expression of EAAT2, xCT, and ASCT1 in the epilepsy group, while no distinct regulation was found for CD98 (fold change 0.07) (Fig. 2D). In GB patients with epilepsy, we found 120 proteins significantly downregulated, and 94 proteins to be significantly upregulated (Fig. 2E). Of the top 20 proteins with the highest fold increase, seven were encoded by genes in which mutations can cause epilepsy, namely SLC6A11, SLC6A9, EAAT2 (*SLC1A2*), SLC4A4, TMLHE, FGF1, and WFS1 [19] (supplementary Table 1). In the protein enrichment analysis, we found that Gene Ontology-Biological Process (GO-BP) terms, such as amino acid neurotransmitter reuptake and neurotransmitter reuptake, were the clusters with the highest fold increase (Fig. 2F). In addition, amino acid metabolism was indicated in other GO-BP terms, such as processing of carboxylic acids. Furthermore, we detected a significant enrichment of GO-BP terms involving lipid metabolism. The top 15 enriched GO-BP terms comprised 54 proteins (Fig. 2G), among which were several members of the solute carrier (SLC) family. These include EAAT2, as well as proteins involved in amino acid metabolism, such as the 4-aminobutyrate aminotransferase (ABAT), or proteins facilitating ion transport across the plasma membrane, such as the ATPase Na+/K+ transporting subunit alpha 2 (ATP1A2), the inositol-trisphosphate 3-kinase B (ITPKB), and the potassium inwardly rectifying channel subfamily J member 10 (KCNJ10). Examples of proteins involved in lipid metabolism were the acyl-CoA synthetase long chain family member 6 (ACSL6), the aldolase C (ALDOC), the acyl-CoA dehydrogenase family member 11 (ACAD11) and the epoxide hydrolase 2 (EPHX2).

### Impact of high xCT expression on the proteome of IDH-wildtype glioblastoma with glioma-associated epilepsy

**Fig. 3:**
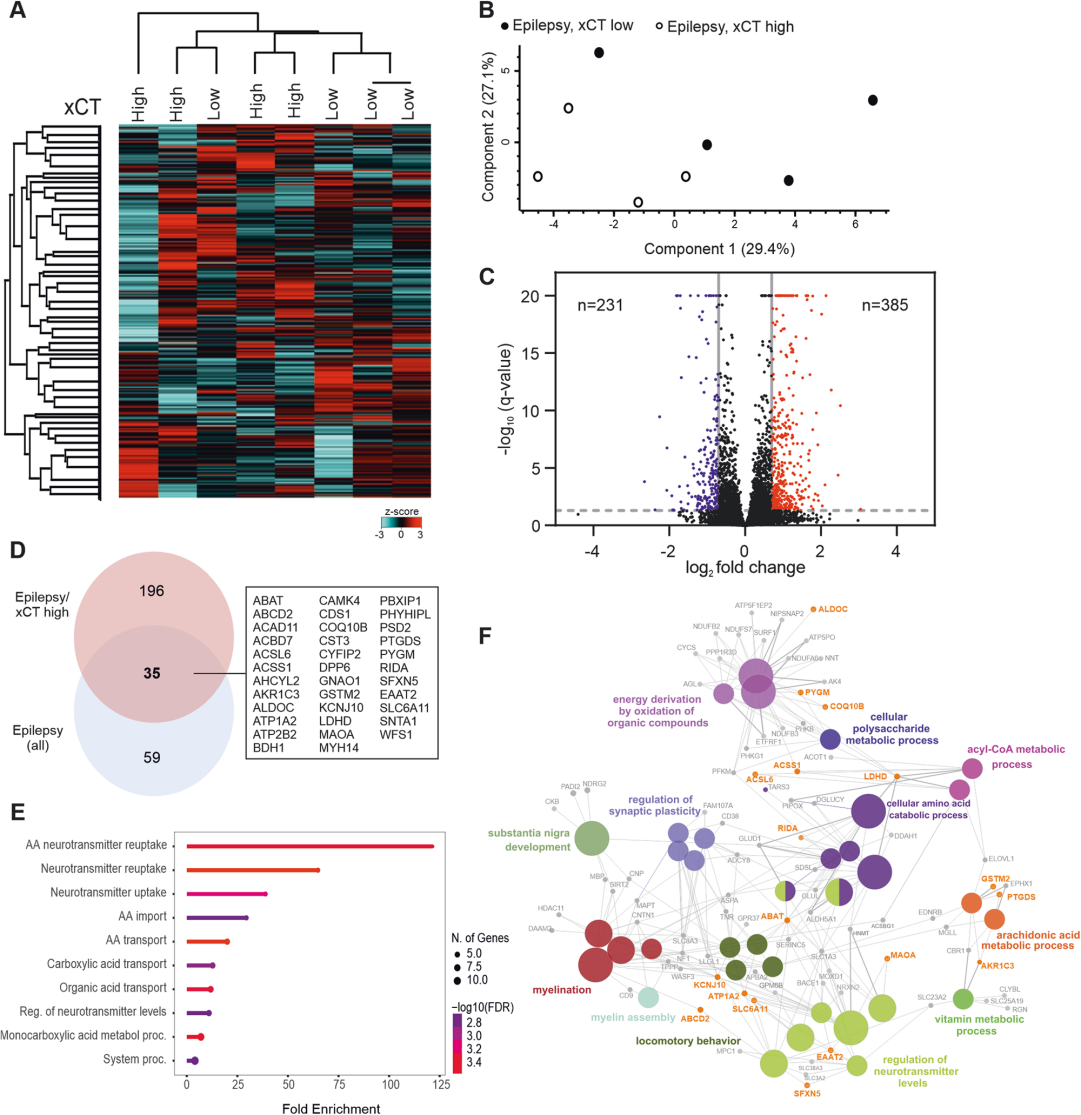
Whole-cell proteomics of IDH-wildtype glioblastoma samples with epilepsy and low versus high xCT expression. **A** Heat map showing row-scaled z-scores for all measured proteins across all samples (*n* = 8). **B** Principal component analysis (PCA) results of the samples. **C** Volcano plot showing fold change versus *q*-value for samples of the epilepsy group with low vs. high protein expression of xCT. Numbers indicate the total amount of significantly regulated proteins (*q*-value < 0.05, fold change ≤ −0.07 or ≥0.07). *Q*-values > 20 were set to *q* = 20 for plotting; for original *q*-values, please refer to supplementary Table 1. **D** Venn diagram showing the overlap of significantly upregulated proteins between cases with epilepsy and cases with epilepsy and high xCT expression. **E** Protein enrichment analysis of overlapping proteins from (**D**) (*q*-value < 0.05, fold change ≥ 0.07); displayed are the top 10 GO-BP terms (*q*-value < 0.05), pathways are sorted by fold enrichment. **F** Representation of the GO-BP network of significantly upregulated proteins in the epilepsy/xCT high-expressing cases; proteins overlapping with the total epilepsy cohort (shown in (**D**)) are highlighted in yellow.

Next, we asked whether the proteome of tumors in the epilepsy group was impacted by xCT expression levels. To this end, we compared the tumors with low xCT expression to high expression within the epilepsy group (Fig. 3). As before (Fig. 1D), classification as either low- or high-expressing tumors was based on the median expression level of the entire cohort. In both the principal component analysis and unsupervised clustering, we observed a distinction between the xCT low- and high-expressing samples (Fig. 3A, B). We found 616 proteins to be significantly different between the two groups (Fig. 3C). There was an overlap of proteins that were upregulated both in the entire epilepsy cohort and in the epilepsy tumors with high xCT expression. This overlap included 35 proteins (Fig. 3D), five of which (ABAT, ALDOC, ACSL6, MAOA, PYGM) were among the top ten significantly regulated proteins of the proteome dataset. Subjecting the 35 proteins to enrichment analysis, we found that they clustered for similar GO-BP terms as proteins upregulated in the epilepsy cohort vs. control (Fig. 2), in particular GO-BP terms related to amino acid and neurotransmitter metabolism as well as lipid metabolism (Fig. 3E). In the overall analysis of proteins that were upregulated in the xCT high-expressing tumors with epilepsy, the enrichment analysis also revealed GO-BP terms beyond neurotransmitter metabolism (Fig. 3F). These included myelination, regulation of synaptic plasticity, substantia nigra development, as well as locomotor behavior.

### Impact of glutamate transporter expression on the clinical outcome of glioma patients

**Fig. 4:**
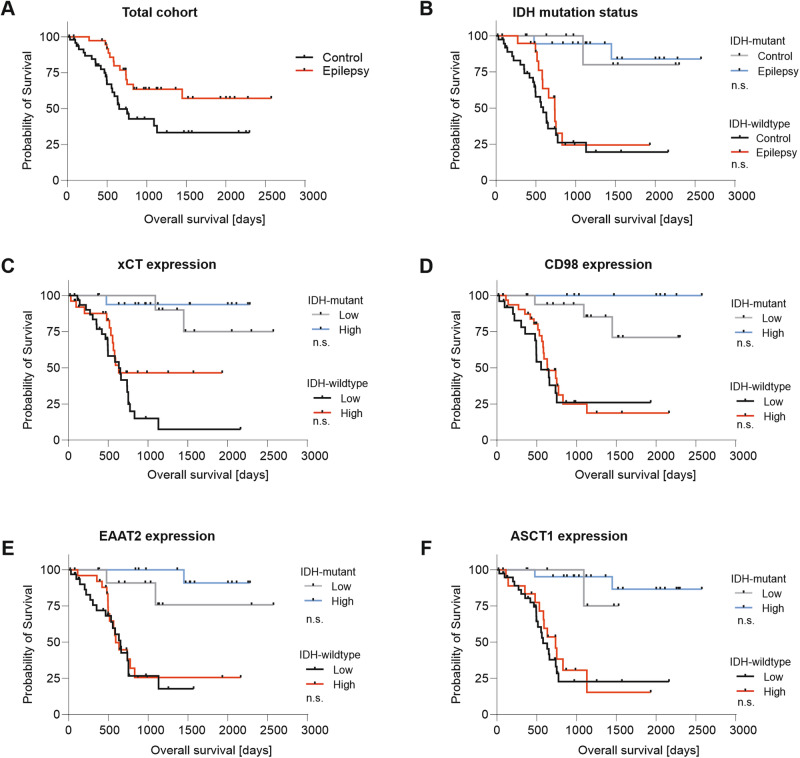
Analysis of overall survival (OS). Impact of epilepsy on OS of **A** the total cohort (*n* = 87) and **B** patients with tumors bearing or lacking IDH mutation, as indicated. Impact of protein expression levels of **C** xCT, **D** CD98, **E** EAAT2, and **F** ASCT1 on OS of IDH-wildtype GB (*n* = 62). Tick marks indicate censored patients. Abbreviations: n.s. not significant (log-rank test).

Our immunoblot and proteome data demonstrate that altered expression of glutamate and amino acid transporters in gliomas derived from patients with epilepsy at first diagnosis is common. As it has been shown that glutamatergic neuron-to-glioma synapses drive the progression of gliomas by stimulating cell invasion and growth [7], we investigated the association of seizures with outcome in our cohort. In the total cohort, overall survival (OS) was significantly improved in patients with epilepsy as compared to the non-epilepsy group (hazard ratio 0.48, 95% CI 0.26–0.89, *p* = 0.02 log-rank test) (Fig. 4A). However, OS did not differ between epilepsy and control cases in either IDH-wildtype (i.e., GB) or IDH-mutant tumors (Fig. 4B). This suggests that the improved OS of the epilepsy cohort is confounded by its high percentage of IDH mutant tumors. Lastly, we examined the impact of protein expression levels of the four transporters we had investigated by immunoblot. To this end, cases were again dichotomized as either low- or high-expressing based on the median expression level of the entire cohort. For none of these proteins did the expression level have a significant impact on the overall survival of IDH wild-type or IDH-mutant gliomas. (Fig. 4C–F).

## Discussion

In this study, we conducted a comparative analysis of four transporters central to the regulation of extracellular glutamate levels. Among these, only ASCT1 and EAAT2 reached statistical significance in the immunoblot analysis. This finding was corroborated by mRNA data from IDH-mutant gliomas. In IDH-wildtype gliomas, elevation of EAAT2 and ASCT1 was present, but statistically not significant, possibly due to the smaller sample size of this cohort. In the contingency analysis, ASCT1 had the strongest association with epilepsy in the contingency analysis. Moreover, we observed that 83% of IDH-mutant tumors—which generally have a higher incidence of seizures than IDH-wildtype GB—showed high expression of ASCT1. In a recent study based on RNA-seq data from diffuse high-grade glioma, *SLC1A4*, which encodes ASCT1, was identified among the genes with the highest ability to predict postoperative seizures [18]. Together, these observations suggest that ASCT1/*SLC1A4* could be a biomarker for GAE. From a clinical point of view, it should be noted that, in contrast to xCT and EAAT2, no approved inhibitor of ASCT1 exists.

The upregulation of EAAT2 may appear contradictory, as the elimination of glutamate from the extracellular space is the main function of EAAT2. Indeed, our findings contrast with findings from mouse models and a retrospective study of glioma tissue [20, 21]. Moreover, they differ from a study of 25 IDH wildtype gliomas that reported no distinct mRNA expression of EAAT2 in the epilepsy group [22]. However, it has also been described that EAAT2 expression is dynamically adapted to high extracellular concentrations of glutamate [23]. Hence, the high expression of EAAT2 in our epilepsy cohort, as well as the mRNA datasets, could represent a compensatory mechanism. This aspect warrants validation in larger cohorts.

Although xCT has been studied most extensively as a pharmacological target for ASM, the association of high xCT expression with seizures was present but less robust compared to EAAT2 and ASCT1. This may be due to the sample size on the one hand, and to both intra- and inter-tumor heterogeneity on the other, as reported previously [24]. In this context, it should also be noted that the occurrence of seizures in our cohort was not exclusive to cases with high xCT expression, indicating drivers of epileptogenesis that are xCT-independent and possibly glioma subtype-specific, such as the hyperexcitability induced by D-2-hydroglutarate (2-HG) in IDH mutant gliomas [25–27] or alterations of ion channels [7]. The observation that CD98 was inversely expressed to xCT has to be interpreted with caution, as CD98 serves as a regulatory subunit for other transporters [28].

The overall increase in transmembrane glutamate activity in GAE found by immunoblot and mRNA analysis was corroborated by our proteome analysis. Moreover, this observation is in line with RNA expression data published recently from a retrospective multicenter analysis of GB [29]. In this study by Ricklefs et al., GB of methylation subclass RTK II had a higher incidence of pre- and post-operative seizures and showed upregulation of genes mediating vesicle transport and neurotransmitter synapses, including members of the synaptic vesicle protein 2 (SV2) and the solute carrier 17 (SLC17) family. The corresponding proteins of these genes were upregulated in the epilepsy cohort of our proteome dataset, thus confirming the robustness of our findings. Similar results have been obtained from RNA expression analysis of low-grade gliomas [30, 31], implying that different glioma entities may share pathomechanisms of epileptogenesis.

Despite the fact that the association of xCT with seizure occurrence was inferior to EAAT2 and ASCT1, the distinct proteome pattern observed in xCT high-expressing cases underlines how xCT expression levels can shape the biological network of gliomas. More specifically, the overlap with the total epilepsy group of proteins linked to neurotransmitter and amino acid metabolism may indicate that these enrichment clusters are driven by high xCT expression, which underlines its central role in the regulation of glutamate in glioma cells. In addition to neurotransmitter and amino acid processing, we found proteins linked to lipid metabolism to be upregulated in both the epilepsy cohort and the xCT high-expressing tumors. Disorders of lipid metabolism have been identified as pathogenic drivers of epilepsy and may affect all classes of lipids, including triglycerides and fatty acids [32]. Mechanistically, neuron hyperactivity has been described to alter lipid metabolism by causing dysfunctional lipid uptake and oxidation, which may ultimately lead to accumulation of lipids in astrocytes, amplify neuronal hyperexcitability [33] and facilitate glioma cell survival [34].

Despite their involvement in transmembrane signaling, the impact of glutamate transporter expression on the prognosis of patients remains unclear. While high xCT expression has been associated with shorter survival in animal and in silico models of glioma [24], it influenced neither the survival of glioma patients in our cohort nor that reported in other studies [35]. This aspect must therefore be clarified in larger cohorts.

## Conclusion

In summary, our results indicate a distinct regulation of glutamate homeostasis in GAE across both IDH mutant and IDH wild-type gliomas. Given the role of glutamate as an excitatory neurotransmitter, a causal link between dysregulation of its transporter network and GAE is biologically plausible. Moreover, our results also show that the protein network of gliomas is shaped by both GAE and xCT expression levels. Our proteomic dataset of gliomas, stratified by the presence of GAE and defined xCT levels, represents a valuable resource for future investigations.

### Limitations

The main limitation of our study is the relatively small sample size, which reduces the statistical power and limits the generalizability of the findings. This limitation particularly affects the proteomic analysis, which is constrained by the relatively small sample size in the subcohorts. Nevertheless, the observed associations between the investigated proteins and epilepsy are biologically plausible and corroborated by other studies. Additional investigations incorporating larger well-powered cohorts will be essential to validate and extend our findings.

## Methods

### Study cohort

Snap-frozen glioma tissue was obtained from resected tumor tissue. Tumors were diagnosed at the Goethe University Frankfurt, University Hospital, Neuropathology Department (Edinger Institute) by at least two experienced neuropathologists according to the 2021 WHO classification of CNS tumors [36]. Formalin-fixed paraffin-embedded (FFPE) glioma tissue was subjected to DNA isolation followed by bisulfite conversion. The samples were prepared for the Human Methylation EPIC array (Illumina, San Diego, USA) covering 850 000 CpG sites according to the manufacturer’s recommendations. Epigenetic molecular GB subclasses were determined by the brain tumor classifier version 11b4 provided by the molecularneuropatholog.org platform. Prior to further analyses, frozen tissue samples were analyzed by HE staining. Samples containing vital tumor tissue were selected, while samples containing necrotic areas or the tumor infiltration zone were excluded. We aimed for a tumor cell content of ≥70% of the samples as assessed by microscopy. Cases of tumor recurrence were excluded from the final cohort.

Seizures and epilepsy were diagnosed according to the latest definitions established by the International League Against Epilepsy (ILAE) [37, 38].

### Cell lines and murine brain slices

H4, LN-229 and T98G cells were purchased from the American Type Culture Collection (ATCC). H4 xCT knockout cells and brain tissue derived from xCT-deficient and wild-type mice [39] were provided by UCB Pharma (Brussels, Belgium).

### Immunoblot and immunohistochemistry

Tumor tissue was homogenized in RIPA buffer containing protease/phosphatase inhibitor at 50 Hz for 5 min. After a 15-min incubation on ice, the samples were centrifuged at 13,000 rpm for 5 min. Protein concentration of tissue samples and cell lines was determined using a Bradford protein assay (BioRad, Munich, Germany). We used Laemmli buffer with dithiothreitol (DTT) as a loading buffer. We subjected 10 µg protein to SDS page analysis. After washing with tris-buffered saline with Tween20 0.1% and blocking in 5% skim milk, membranes were incubated overnight with antibodies to EAAT2 (rabbit monoclonal, Abcam 178401, working dilution 1:1000), 4F2hc/CD98 (rabbit monoclonal, Cell Signaling #13180, 1:1000), ASCT1 (rabbit polyclonal, Bethyl Laboratories #A305-283, 1:1000), xCT (rabbit monoclonal, Cell Signaling #12691, 1:1000) or actin (goat polyclonal, Santa Cruz sc-1616, 1:2000). Following one hour of incubation with secondary antibody, we used chemiluminescence for detection. For immunoblot, we used LN229, T98G and H4 wildtype cells as positive controls, and H4 xCT knockout cells as negative controls.

For immunohistochemistry, we used a previously published xCT antibody (rabbit polyclonal, Abcam #ab37185, lot GR3353159-1, 1:250) [35] employing a standard protocol [40].

### Mass spectrometry

Snap-frozen tumor tissue was collected in 1 M Tris/20% SDS. Samples were homogenized at 50 Hz for 5 minutes. Tumor lysates were then precipitated by methanol/chloroform, and proteins were resuspended in Urea/EPPS. We determined the concentration of proteins by Bradford assay and used 50 µg of protein per sample for digestion with 1:50 LysC (Wako Chemicals, Neuss, Germany) and 1:100 sequencing-grade trypsin (Promega, Madison, WI, USA). Digests were purified by tC18 SepPak (50 mg, Waters, Milford, MA, USA). Per sample, we labeled 25 µg peptides with TMTpro and the mixing was normalized after a single injection measurement by LCMS/MS to equimolar ratios for each channel.

### Offline high pH reverse phase fractionation

Peptides were fractionated using a micro-flow HPLC (Dionex U3000 RSLC, ThermoFisher Scientific, Waltham, MA, USA). Totally, 45 µg of pooled and purified TMT labeled peptides resuspended in Solvent A (5 mM ammonium-bicarbonate, 5% ACN) were separated on a C18 column (XSelect CSH, 1mm × 150mm, 3.5 µm particle size; Waters) using a multistep gradient from 3 to 60% Solvent B (5 mM ammonium-bicarbonate, 90% ACN) over 65 min at a flow rate of 30 µl/min. Eluting peptides were collected every 43 s from minute 2 for 69 min into a total of 96 fractions, which were cross-concatenated into 24 fractions. Pooled fractions were dried in a vacuum concentrator and resuspended in 2% ACN, 0.1% TFA for LC–MS analysis.

### Liquid chromatography mass spectrometry

All mass spectrometry data were acquired on an Orbitrap Fusion Lumos mass spectrometer (ThermoFisher Scientific, Waltham, MA, USA) equipped with a nanoFlex ion source (Thermo Fisher Scientific). To separate peptides, we used a self-made, 22 cm long, 75 µm ID fused-silica column, packed in house with 1.9 µm C18 particles (ReproSil-Pur, Dr. Maisch, Ammerbuch-Entringen, Germany) kept at 50 °C using an integrated column oven (Sonation). For proteome analysis, individual peptide fractions were eluted by a non-linear gradient from 7 to 40% B followed by a step-wise increase to 75% B in 6 min which was held for another 9 min.

Full scan MS spectra (350–1400 m/z) were acquired with a resolution of 120,000 at m/z 200, maximum injection time of 100 ms and AGC target value of 4 × 105. The 10 most intense precursors with a charge state between 2 and 6 per full scan were selected for fragmentation and isolated with a quadrupole isolation window of 0.7 Th. MS2 scans were performed in the Ion trap (Turbo) using a maximum injection time of 50 ms, AGC target value of 1.5 × 104 and fragmented using collision-induced dissociation (CID) with a normalized collision energy (NCE) of 35%. SPS-MS3 scans for quantification were performed on the 10 most intense MS2 fragment ions with an isolation window of 0.7 Th (MS) and 2 m/z (MS2). We used HCD with an NCE of 50% to fragment ions and analyzed them in the Orbitrap with a resolution of 50,000 at m/z 200, scan range of 100–500 m/z, AGC target value of 1.5 × 105 and a maximum injection time of 86 ms. Repeated sequencing of already acquired precursors was limited by setting a dynamic exclusion of 45 s and 7 ppm, and advanced peak determination was deactivated.

### Mass spectrometry data analysis

Raw files were analyzed using Proteome Discoverer (PD) 2.4 software (ThermoFisher Scientific). Spectra were selected using default settings, and database searches were performed using the SequestHT node in PD against the Human reference proteome (HoSP_OSPG_20230214.fasta). Trypsin (full) was specified as the digestion enzyme with a maximum of 2 missed cleavage sites allowed. Dynamic modifications were TMTpro ( + 304.207 Da) at the N-terminus and lysine residues, acetylation (Protein N-terminus, +42.011 Da), and Oxidation ( + 15.995 Da). Static modifications included carbamidomethyl at cysteine residues ( + 57.021 Da). Precursor mass tolerance was set to 7 ppm and fragment mass tolerance to 0.5 Da. False discovery rates were controlled using Percolator (<1% FDR on PSM level). Normalized PSMs were summed for each peptide as described previously [41] and data exported for further use.

For principal component analysis (PCA) and unsupervised clustering, we used Perseus software 2.0.11. For protein enrichment analysis, we used Cytoscape software 3.10.2 [42] with CluegoApp 2.5.10 [43] and stringApp 2.1.1 [44], as well as ShinyGO (http://bioinformatics.sdstate.edu/go/). For protein enrichment analysis, we used a cut-off of 0.7 and −0.7 for fold change and a *q*-value of <0.05 for significance.

### Analysis of transcriptomic datasets

Two public datasets were analyzed to assess *SLC1A2, SLC1A4, SLC7A11*, and *SLC3A2* expression in relation to seizure history. For the TCGA-LGG cohort [45], RNA-seq counts were obtained using the R package TCGAbiolinks (v2.30.4; [46]) and restricted to primary tumors with documented seizure history. Differential expression was tested using DESeq2 (v1.42.0; [47]) via the Wald test, with Benjamini–Hochberg adjustment calculated across the four predefined candidate genes; VST-transformed values were used for visualization. For the GSE199759 cohort [48], microarray data comparing glioma with (GRE) vs. without (GNE) epilepsy were retrieved using GEOquery (v2.70.0; [49]). Probes were mapped to gene symbols via biomaRt (v2.58.2; [50]), and probe selection was based on the lowest *P*-value identified by limma (v3.58.1; [51]) analysis. Differences were assessed using the Wilcoxon rank-sum test with Benjamini–Hochberg adjustment.

### Statistical analysis

Unless stated otherwise, values are depicted as mean ± SD. For statistical analysis, we used Microsoft Excel (Microsoft, WA, USA) and GraphPad Prism 10.1.2. (Boston, MA, USA) and JMP Pro 18 (SAS Institute, NC, USA). To test for normal distribution, we used the Shapiro–Wilk test. To test statistical significance between two groups that were non normally distributed, we used the Mann–Whitney *U* test. A value of *p* < 0.05 was considered to be statistically significant (*p < 0.05; **p < 0.01; ***p < 0.001). For the contingency analysis, we used Pearson’s chi-squared test. Survival analysis was conducted with GraphPad Prism 10.1.2. (Boston, MA, USA). OS was defined as the duration between biopsy or resection and death from any cause, with survival curves generated using the Kaplan–Meier method. Comparison between the two cohorts was calculated by the log-rank test.

## Supplementary information

Supplementary Figures

Supplementary Figure 3 - Original Western Blots

Supplementary Table 1

## Data Availability

The mass spectrometry proteomics data were deposited in the PRoteomics IDEntifications (PRIDE) Archive database of the ProteomeXchange (PX) consortium [52] with the identifier PXD062733.
