# Supplementary material for: Tumor-associated epilepsy and high expression of xCT shape the proteome of IDH-wildtype glioblastoma
Source: Cell Death Discov. 2026 Mar 25;12:180. doi: 10.1038/s41420-026-03029-7 (PMC13065815; doi:10.1038/s41420-026-03029-7)

# Divé et al., Tumor-associated epilepsy and high expression of xCT shape the proteome of IDH-wildtype glioblastoma

**Supplementary figures**

**Supplemementary Figure 1.** The antibody against xCT was tested in xCT wildtype and knockout mice as well as in human glioma samples with high xCT protein expression, as assessed by immunoblot. The antibody showed unspecific staining of neurons altered due to tissue preparation or ischemia in both xCT wildtype and knockout tissue; examples of this are marked with arrows.


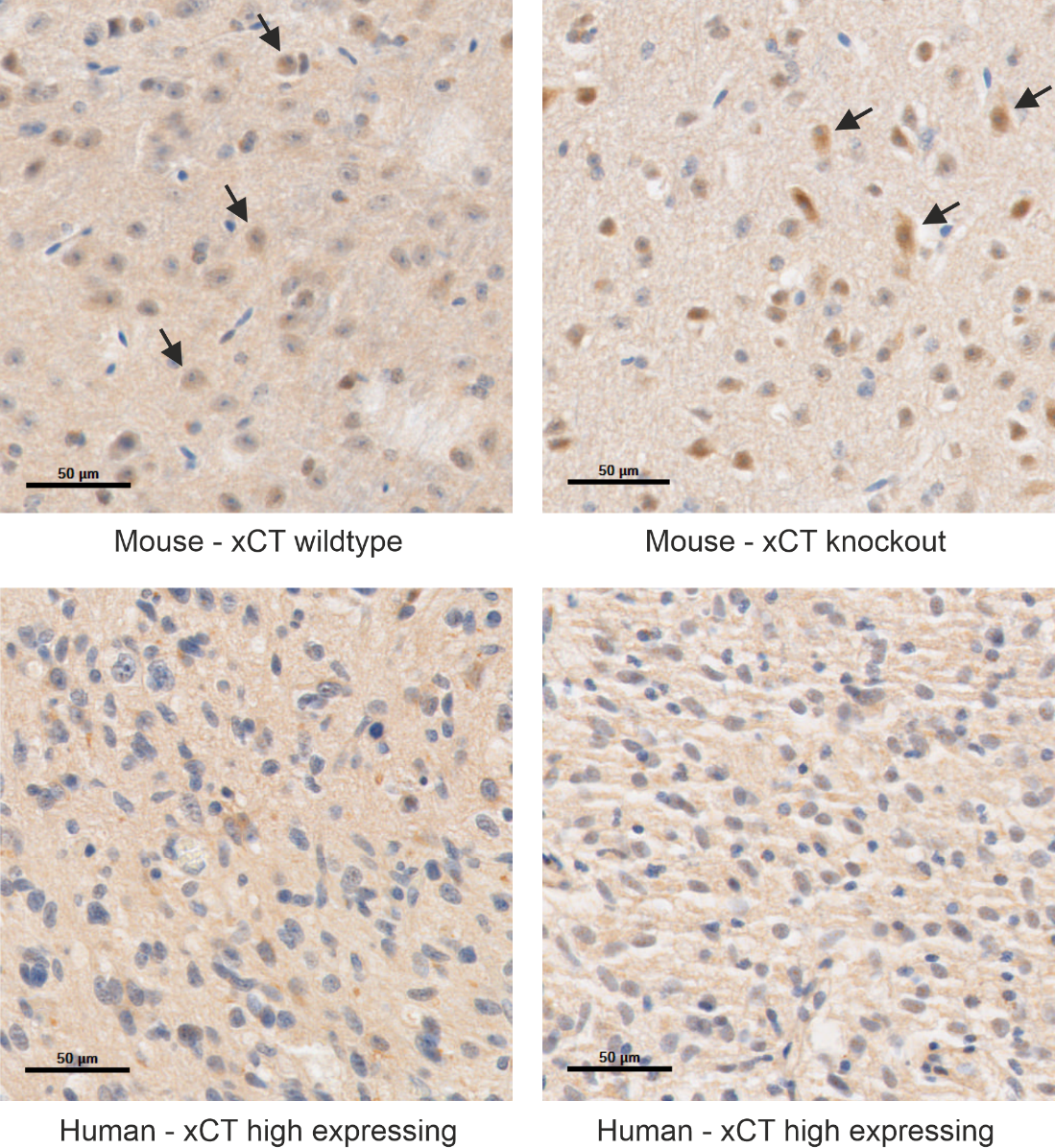


**Supplementary Figure 2**. (A) Protein expression levels of xCT, CD98, EAAT2 and ASCT1 as assessed by immunoblot in IDH-mutant and IDH-wildtype glioma. Values represent protein expression normalized to actin (n=85, bars=mean, error bars=SD; unpaired, two-tailed t-test). (B) Frequency of epilepsy at diagnosis given as percentage of the indicated cohort. (C) Distribution of low and high expression level of the indicated proteins. Tumors were classified as either low- or high-expressing based on the median expression of all samples. Values are given as percentage of the cohort of IDH-mutant or IDH-wildtype gliomas, respectively. (D, E) Comparison of mRNA expression of *SLC7A11*, *SLC3A2*, *SLC1A2* and *SLC1A4* in cases with vs. without epilepsy from the TCGA-LGG dataset (n=480) (D) and from a microarray dataset of IDH wildtype gliomas (n=25) (E). The box spans from the 25th percentile to the 75th percentile, and the horizontal line inside the box represents the median.


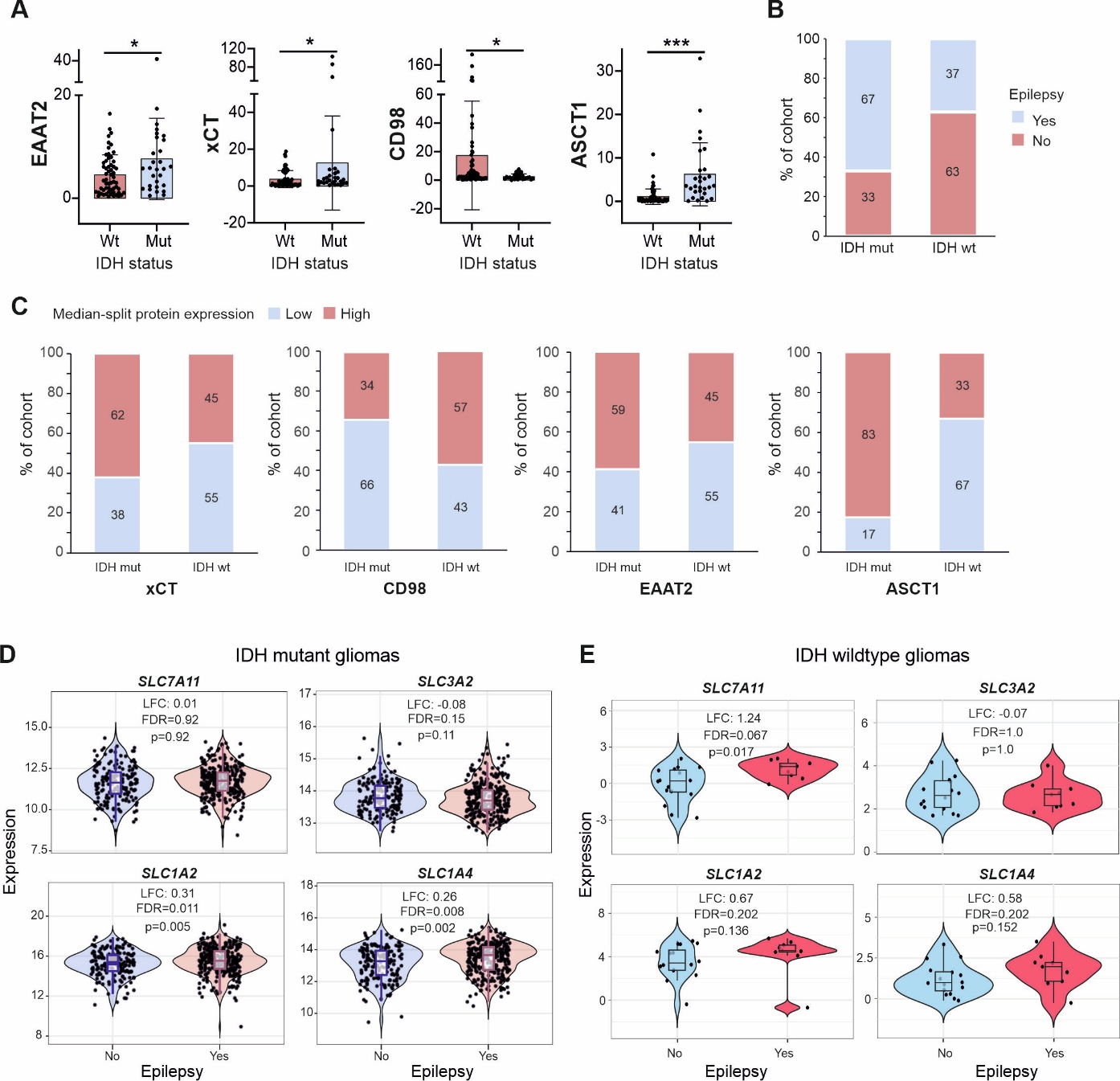

Supplement: Supplementary file 1 — Supplementary Figures [file 41420_2026_3029_MOESM1_ESM.docx]
