# Supplementary material for: Tumor-associated epilepsy and high expression of xCT shape the proteome of IDH-wildtype glioblastoma
Source: Cell Death Discov. 2026 Mar 25;12:180. doi: 10.1038/s41420-026-03029-7 (PMC13065815; doi:10.1038/s41420-026-03029-7)

# Divé et al., Tumor-associated epilepsy and high expression of xCT shape the proteome of IDH-wildtype glioblastoma

**Original Western Blots**

**Supplementary Figure 3.** Uncropped membranes of immunoblots quantifying the protein expression of EAAT2, 4F2hc/CD98, ASCT1, xCT or actin. For xCT, LN229, T98G and H4 wildtype cells were used as positive controls, and H4 xCT knockout cells as negative controls.


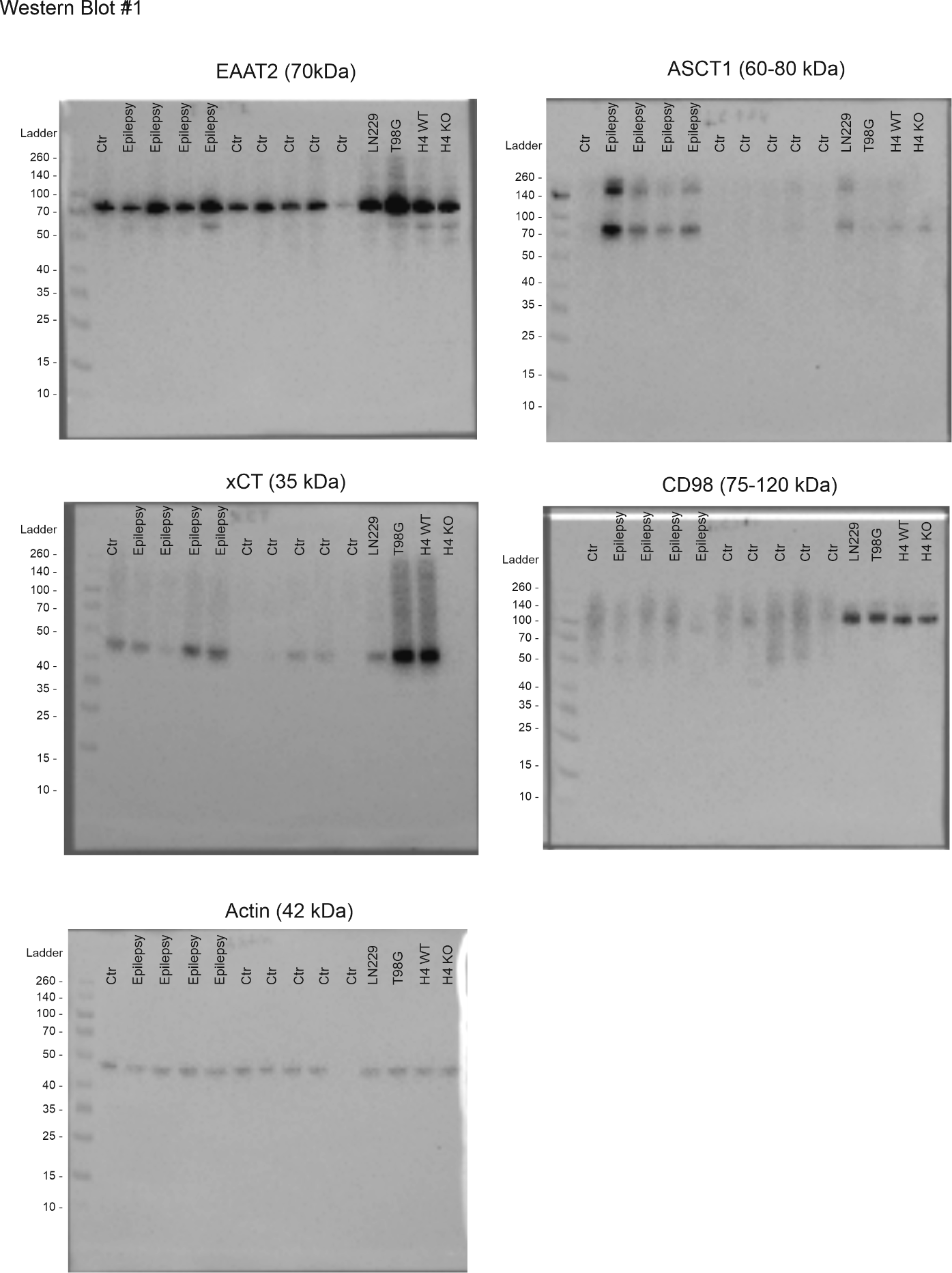


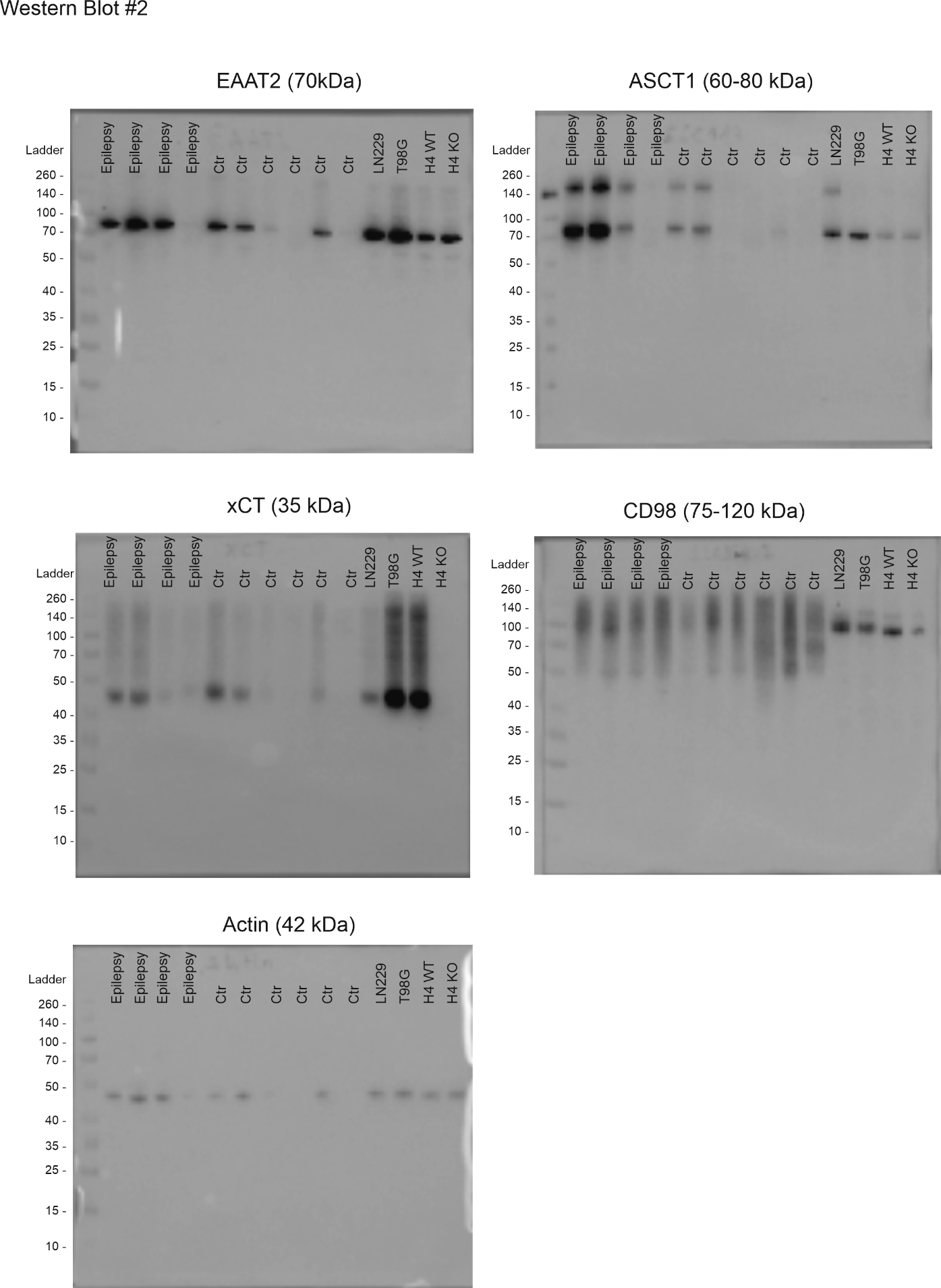

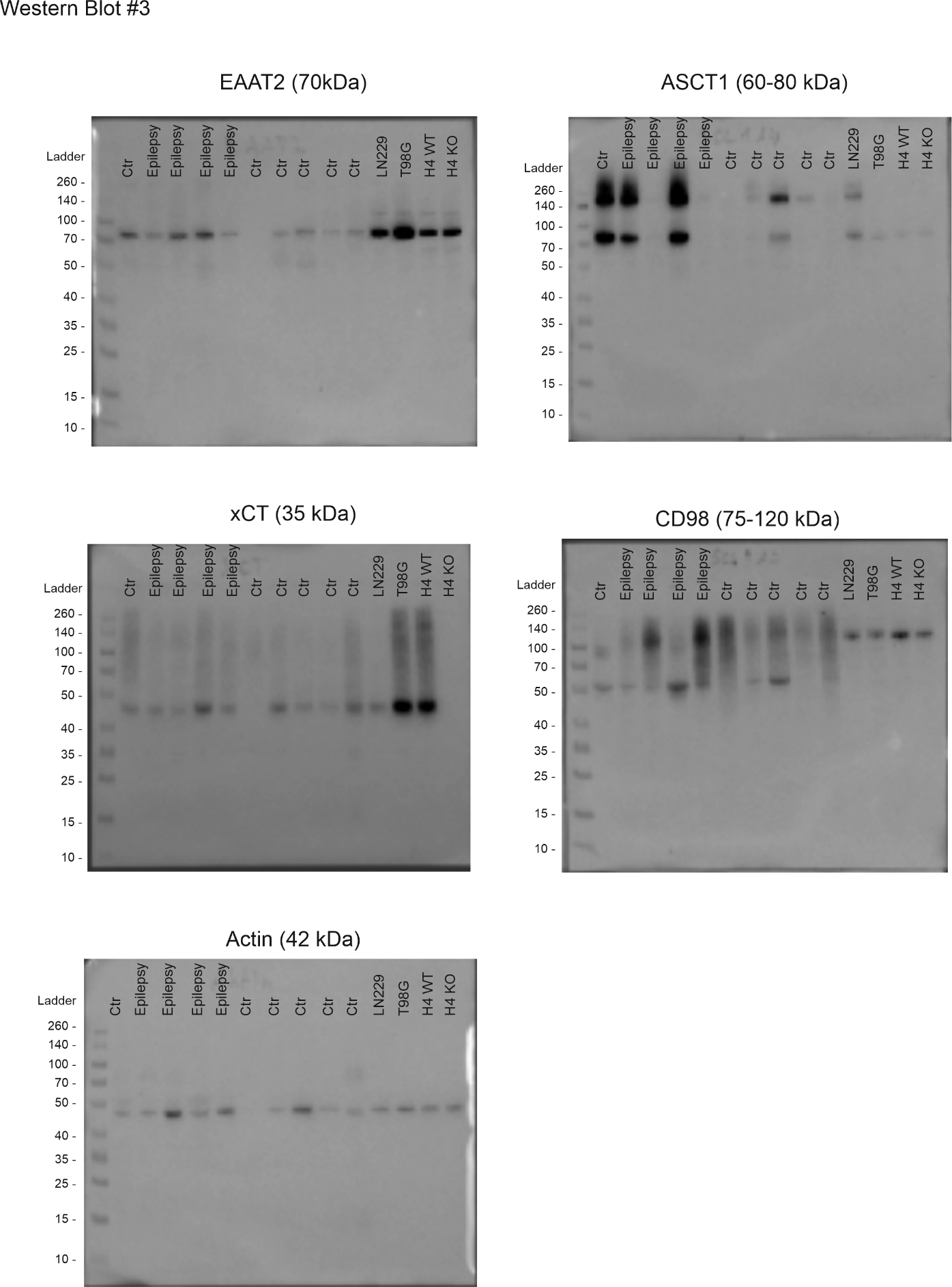

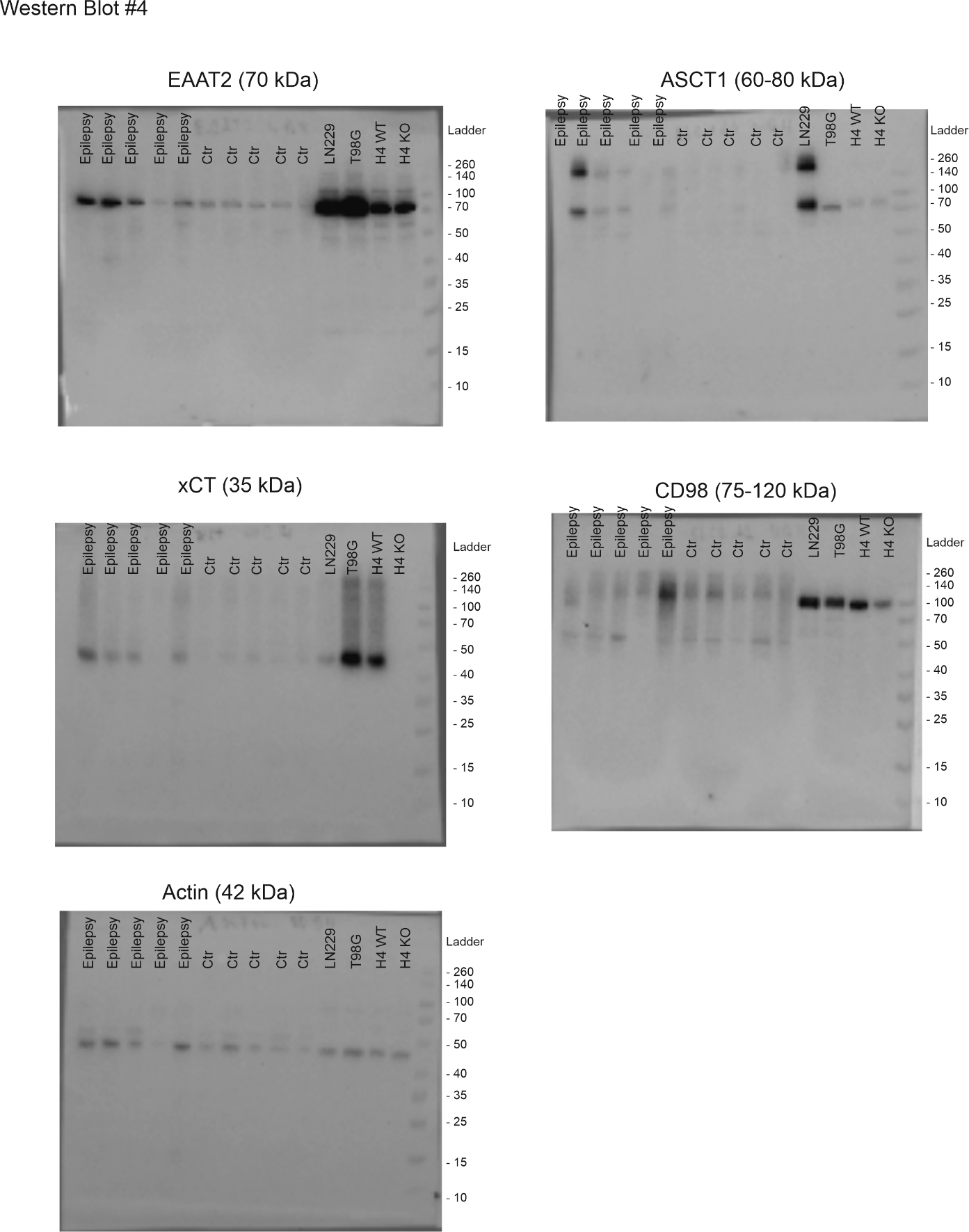

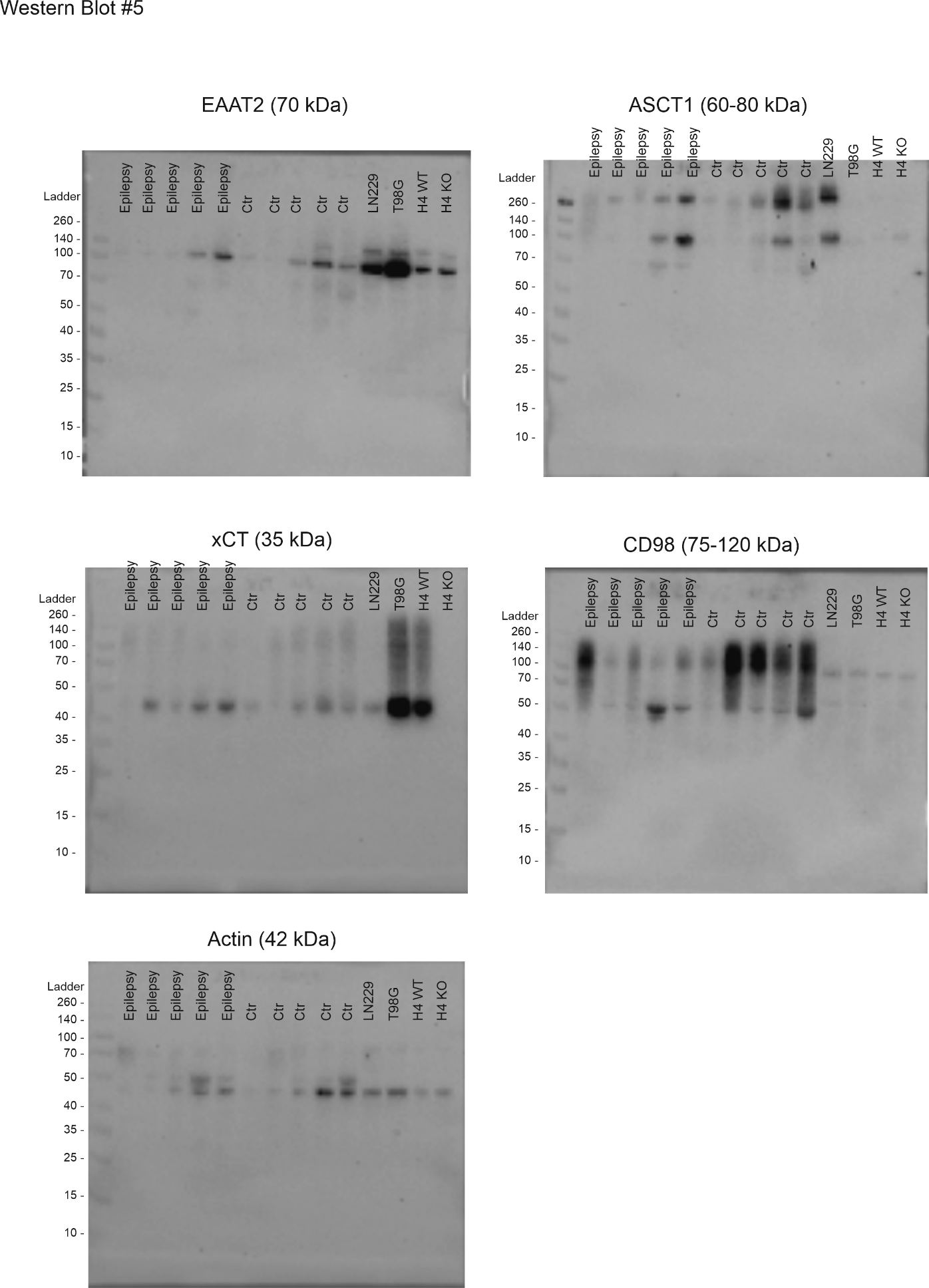

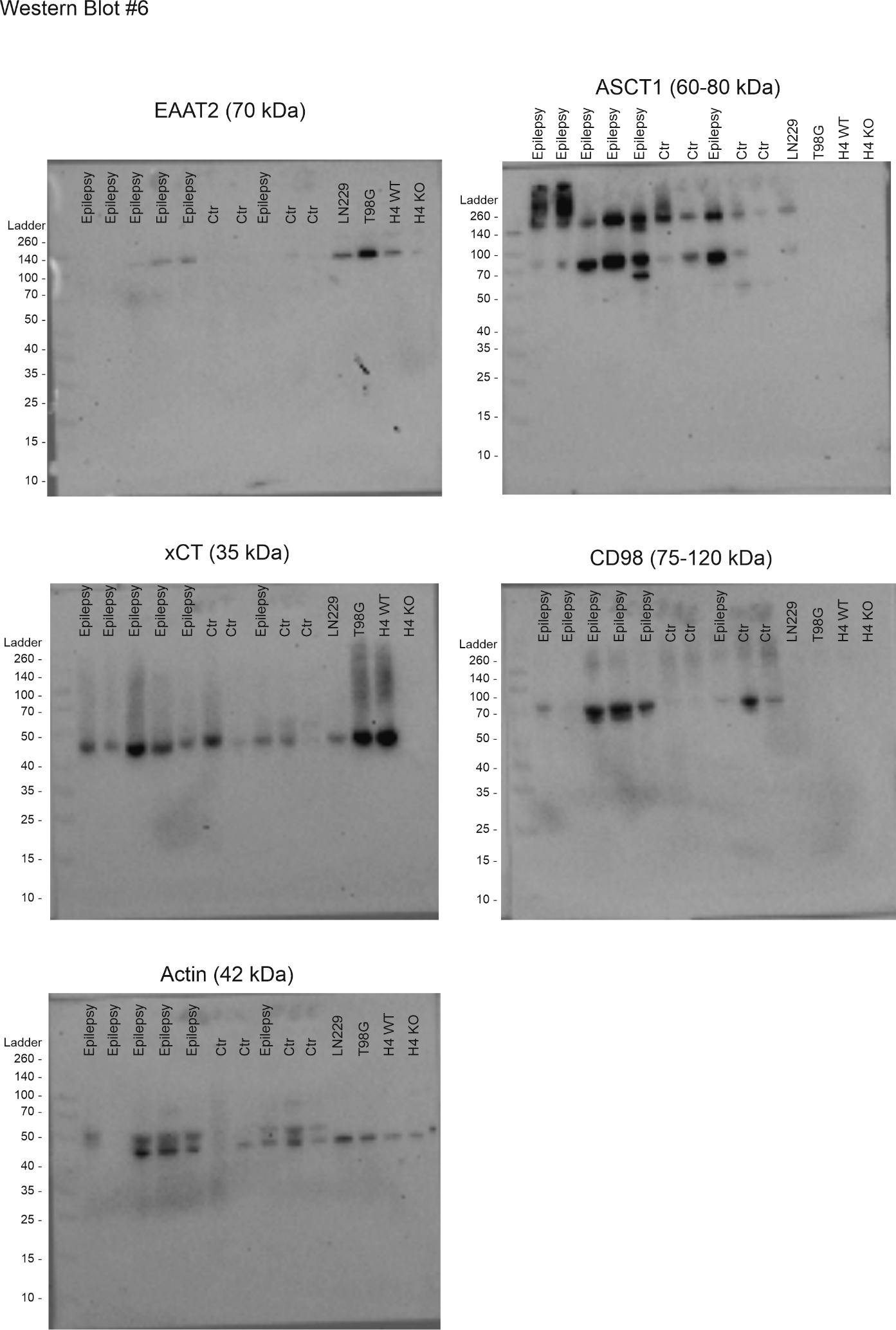

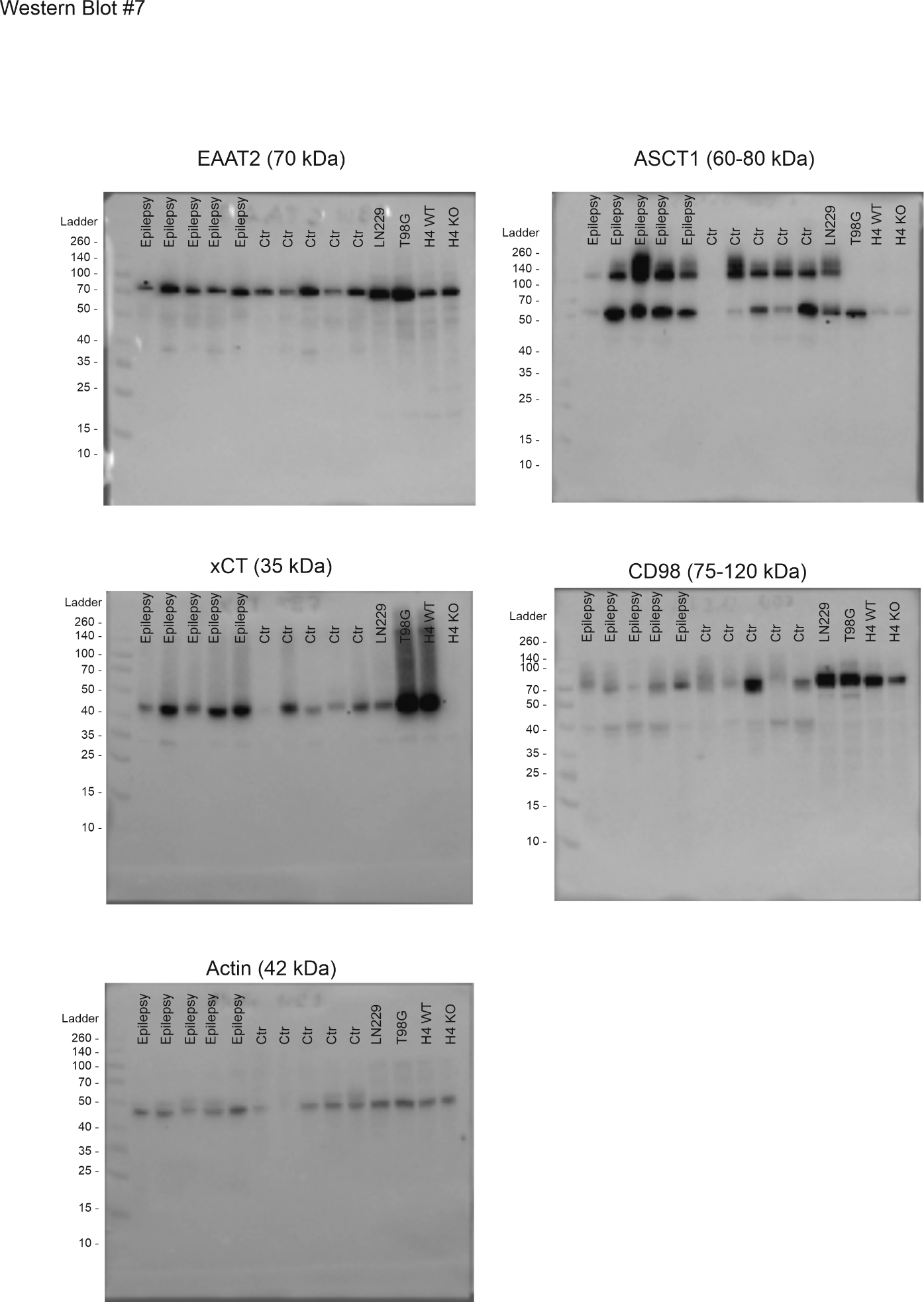

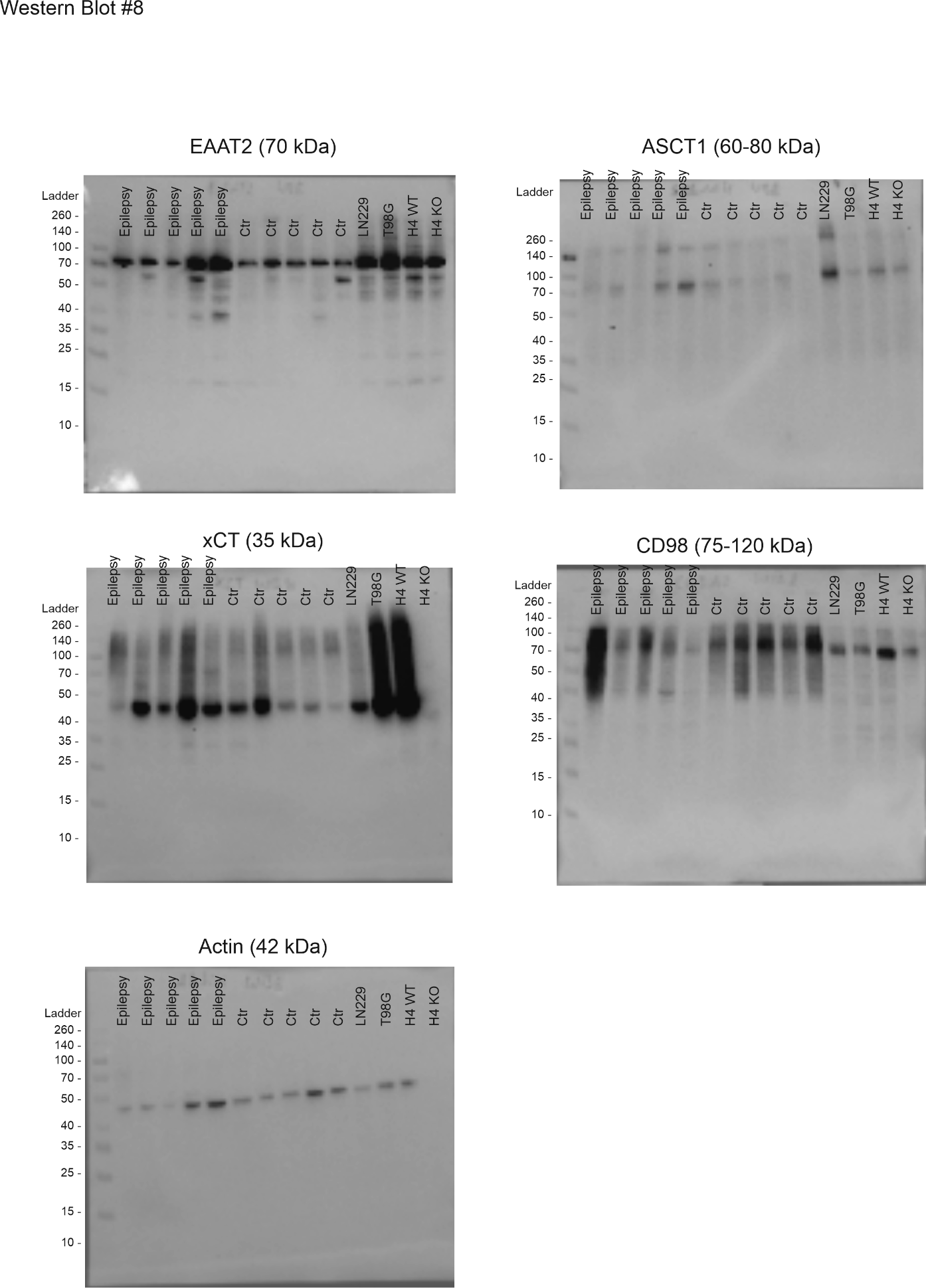

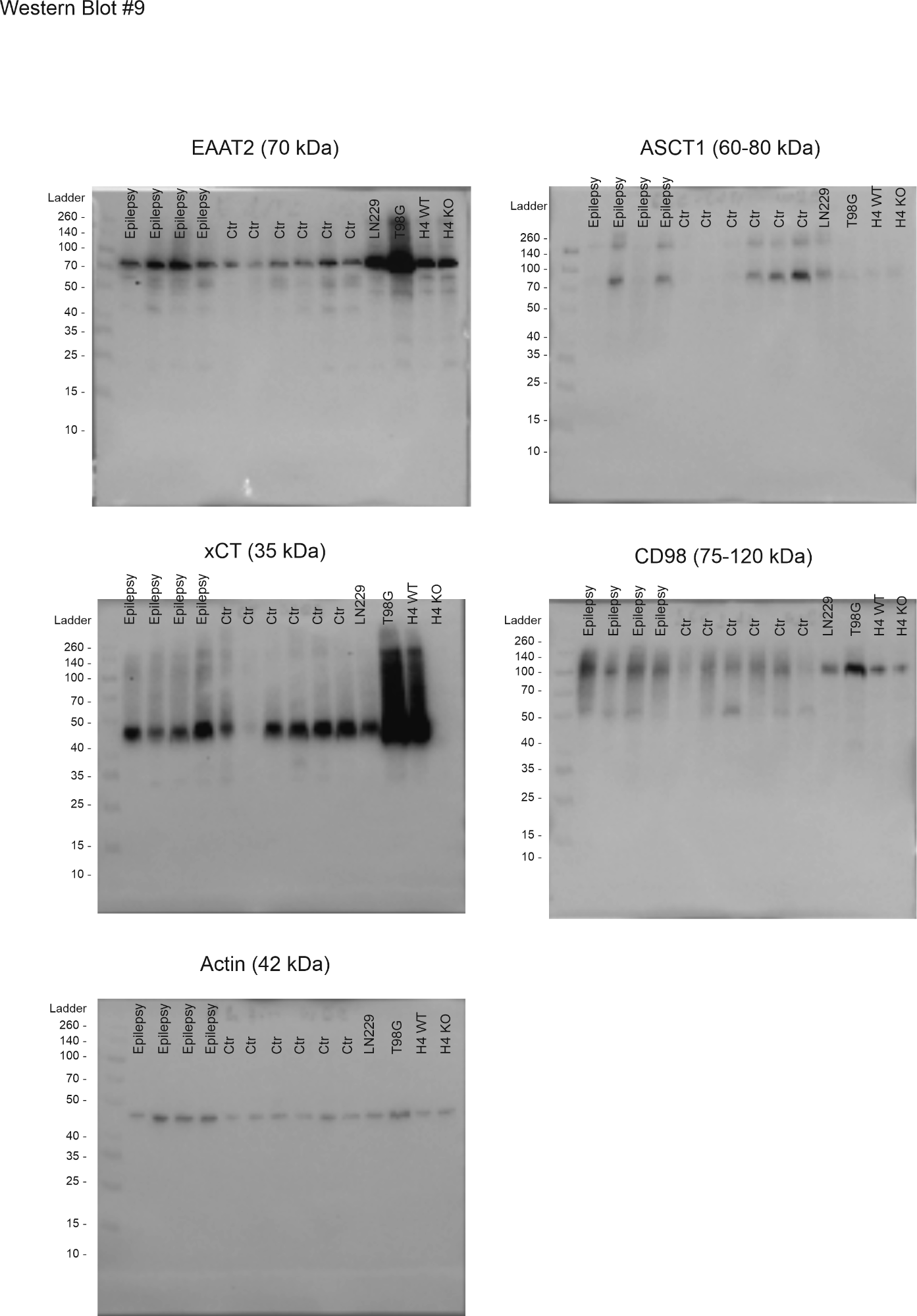

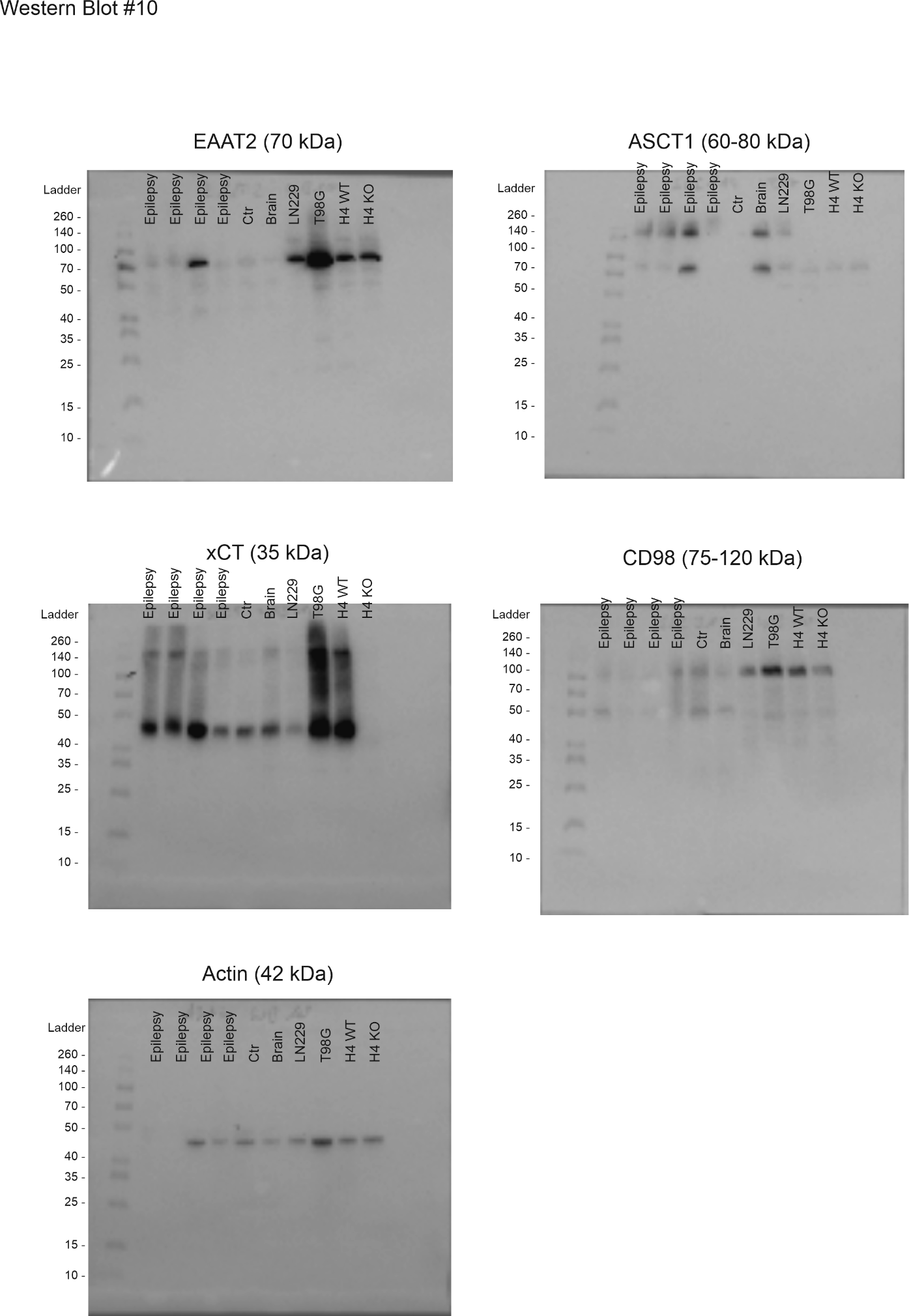

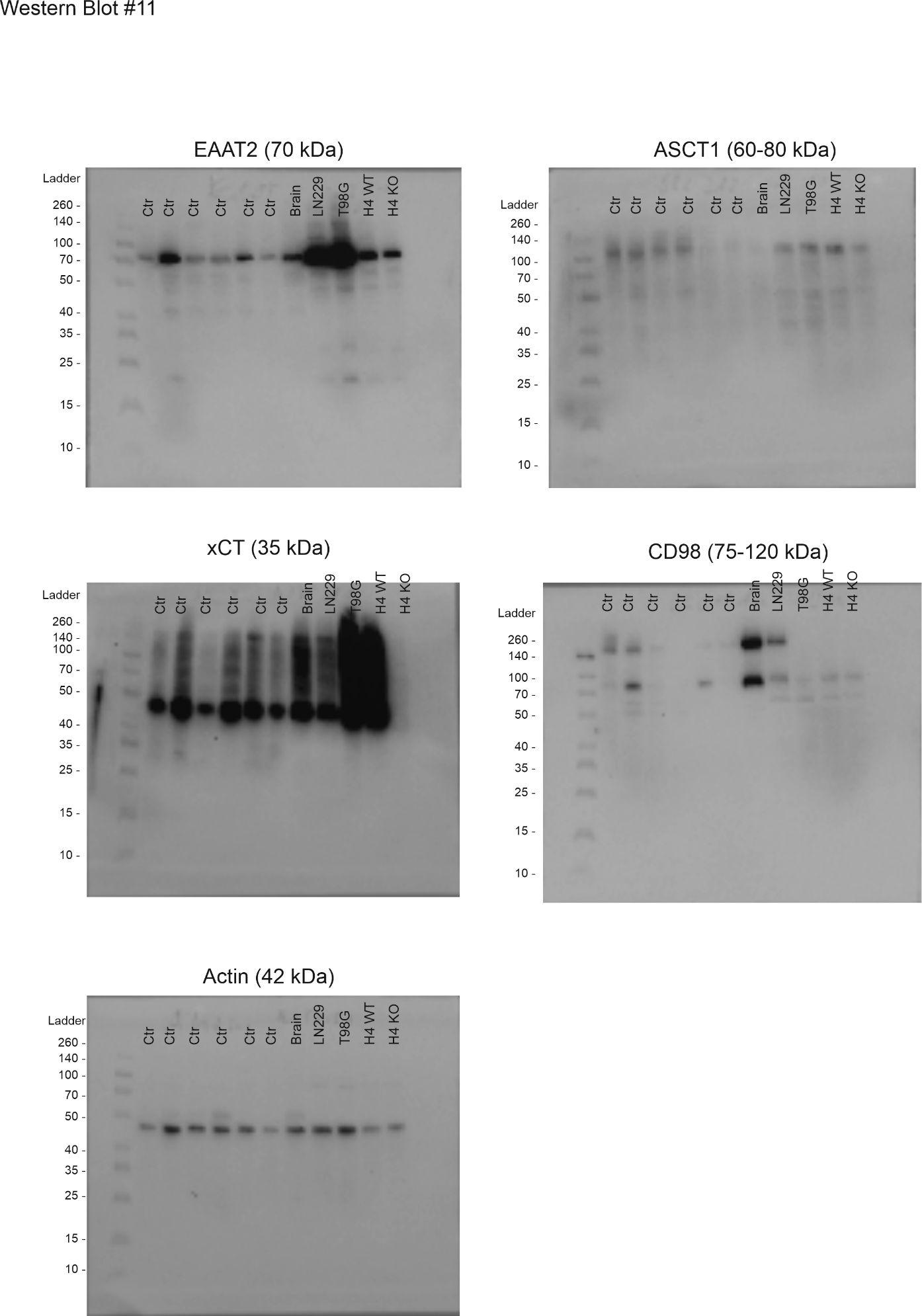

Supplement: Supplementary file 2 — Supplementary Figure 3 - Original Western Blots [file 41420_2026_3029_MOESM2_ESM.docx]
